# Do Lions *Panthera leo* Actively Select Prey or Do Prey Preferences Simply Reflect Chance Responses via Evolutionary Adaptations to Optimal Foraging?

**DOI:** 10.1371/journal.pone.0023607

**Published:** 2011-09-07

**Authors:** Matt W. Hayward, Gina J. Hayward, Craig J. Tambling, Graham I. H. Kerley

**Affiliations:** Centre for African Conservation Ecology, Nelson Mandela Metropolitan University, Port Elizabeth, South Africa; University of California, Berkeley, United States of America

## Abstract

Research on coursing predators has revealed that actions throughout the predatory behavioral sequence (using encounter rate, hunting rate, and kill rate as proxy measures of decisions) drive observed prey preferences. We tested whether similar actions drive the observed prey preferences of a stalking predator, the African lion *Panthera leo*. We conducted two 96 hour, continuous follows of lions in Addo Elephant National Park seasonally from December 2003 until November 2005 (16 follows), and compared prey encounter rate with prey abundance, hunt rate with prey encounter rate, and kill rate with prey hunt rate for the major prey species in Addo using Jacobs' electivity index. We found that lions encountered preferred prey species far more frequently than expected based on their abundance, and they hunted these species more frequently than expected based on this higher encounter rate. Lions responded variably to non-preferred and avoided prey species throughout the predatory sequence, although they hunted avoided prey far less frequently than expected based on the number of encounters of them. We conclude that actions of lions throughout the predatory behavioural sequence, but particularly early on, drive the prey preferences that have been documented for this species. Once a hunt is initiated, evolutionary adaptations to the predator-prey interactions drive hunting success.

## Introduction

African lions *Panthera leo* kill blue wildebeest *Connochaetes taurinus*, Cape buffalo *Syncerus caffer*, gemsbok *Oryx gazella*, giraffe *Giraffa camelopardalis*, and plain's zebra *Equus burchelli* significantly more than expected based on their composition of the prey community at a site [1]. We call this preferential predation, however there is conjecture over whether this involves conscious choice (by hunting in habitats most likely to contain preferred prey or hunting these prey more frequently than expected with random hunting) or is simply a reflection of those species that are easiest to kill because of the morphological adaptations a predator has to hunting them and the responses of prey to these (optimal foraging) [2]. The lion is not unique in exhibiting such preferential predation: African wild dog *Lycaon pictus*, cheetah *Acinonyx jubatus*, and leopard *Panthera pardus* also kill a small number of prey species significantly more frequently than expected, based on their relative abundance in the prey community [3], [4], [5].

Despite this overwhelming evidence of predatory specialization via preferential predation, the question arises whether the predator behaviour drives selection for certain prey species (or individuals) or whether the preferential predation findings arise simply from increased hunting success for those species that the predator has evolved morphological specializations to successfully hunt. Cheetahs do not capture larger prey in more densely vegetated sites, leading to the conclusion that the prey they kill is limited by morphological factors rather than kleptoparasitism avoidance [4]. Similarly, hunting African wild dog packs do not respond to non-preferred prey species when they detect them [6], and the prey preferences in Selous Game Reserve, Tanzania, were reinforced at each stage of the hunting behavioural sequence: encounter rate, decisions on whether to hunt and, ultimately, hunting success [7]. This contrasting predatory behaviour may reflect differences between stalking and coursing predators with coursers having more opportunity to cognitively decide whether or not to hunt.

We aimed to test which actions of a stalking predator – the African lion – influenced whether or not it pursues a particular prey item and whether it captures it. We did this by determining whether lions improved their likelihood of a successful hunt at three distinct phases of the predatory behavioural sequence. Firstly, we determined whether lions encountered preferred prey species more frequently than expected based on the abundance of prey in the community. Secondly, we determined whether lions elected to hunt preferred prey species more frequently than expected based on the encounter rate of prey. Finally, we determined whether lions kill preferred prey more frequently than expected based on the number of attempted hunts. We predicted that lions would 1) encounter preferred prey more frequently than expected in a random encounter rate with prey; would 2) hunt preferred prey more frequently than expected based on random hunts of the encountered prey; and would 3) have a higher hunting success rate on preferred than non-preferred prey species. If these predictions were supported, we believe this would show that lions were making decisions throughout the predator behavioural sequence to optimize their foraging success.

## Results

**Figure 1:**
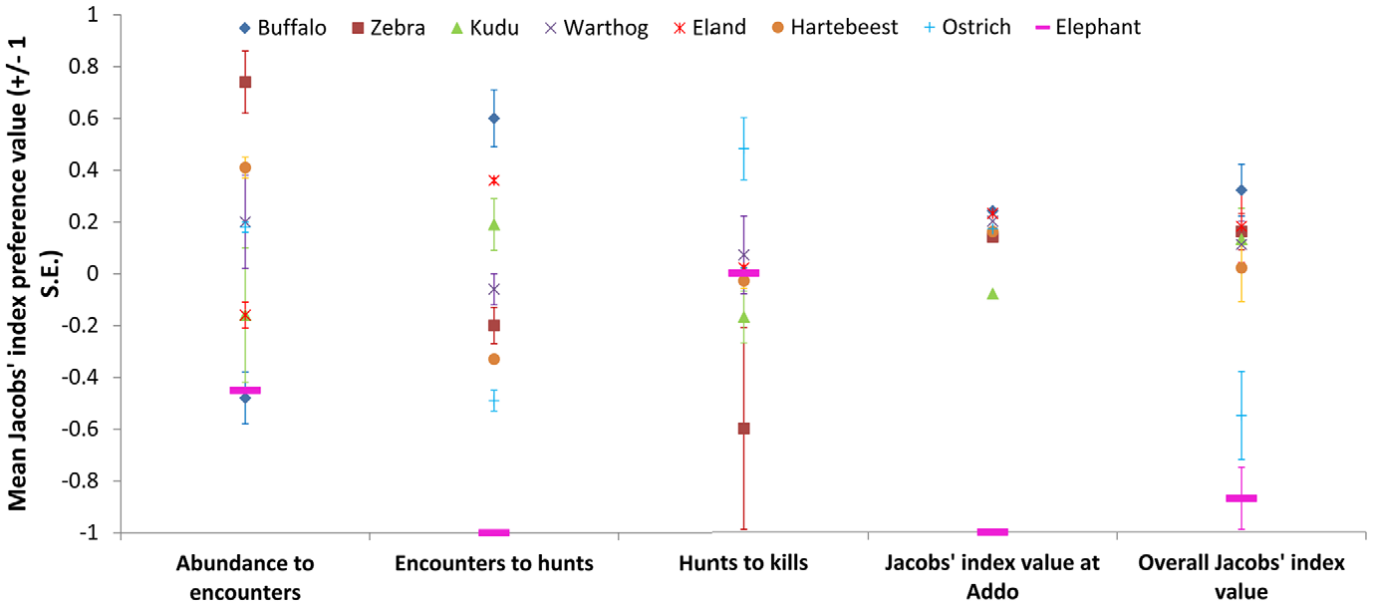
Variation in selectivity (Jacobs' index) for encounters with moving lions, hunts by lions, and kills by lions for the eight most abundant potential prey species in Addo Elephant National Park from December 2003 until November 2005. Overall Jacobs' index values for each species come from the published literature [1]. Jacobs' index values for each species in Addo were calculated using the mean abundance and total number of kills recorded.

**Figure 2:**
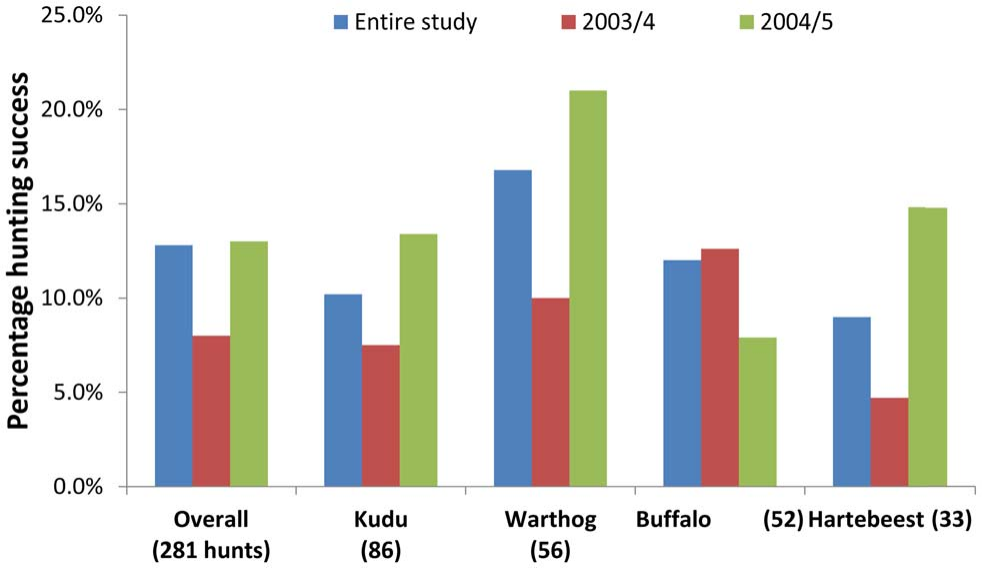
Hunting success (number of kills observed divided by the total number of hunts observed) of lions in Addo Elephant National Park overall, and for the four species that were most frequently observed being hunted.

The buffalo population in Addo exceeded 300 individuals, yet these were encountered by lions far less frequently than expected based on their relative abundance (Fig. 1). This is somewhat misleading, as the buffalo population comprised of ∼ six large herds of up to 100 individuals and approximately ten small bachelor herds and so if we consider the population as 16 herds then we see lions encountered buffalo far more than expected based on their herd abundance (Jacobs' index  = 0.76±0.01). When encountered, the lions hunted the buffalo far more frequently than expected. These hunts led to kills in accordance with their frequency (Fig. 1), with an overall hunting success rate of 11.5% that declined during the second year of the study (Fig. 2). This resulted in preferential predation on buffalo in Addo, in accordance with that throughout the rest of lion range (Fig. 1).

The small zebra population in Addo was encountered by lions far more frequently than expected, based on their relative abundance, and hunts were conducted as frequently as expected based on this high encounter rate (Fig. 1). There were too few successful hunts of zebra in Addo to allow determination of hunting success, however they were preferentially preyed upon in Addo and elsewhere (Fig. 1).

Kudu was the dominant herbivore in Addo, with roughly 1000 present in the park. Lions encountered kudu in accordance with their abundance, they hunted kudu in accordance with their encounter rate, and they killed kudu in accordance with the number of hunts they conducted (Fig. 1). Lions were successful in hunting kudu on 10.5% of hunts, and this success rate improved in the second year of the study (Fig. 2). This resulted in lions non-preferentially hunting kudu in Addo, as they do throughout their range (Fig. 1).

The warthog population in Addo increased from 298 in 2004 to over 600 in 2005. Lions encountered warthogs more frequently than expected based on their abundance, and they elected to hunt the encountered warthogs as expected based on the encounter rate (Fig. 1). Lions converted these hunts into kills as expected based on their frequency (Fig. 1). Lion had a high hunting success rate for warthog (16.1%), and this increased during the second year of the study (Fig. 2). Nonetheless, lions exhibited non-preferential predation on warthog in Addo, as they do throughout their range (Fig. 1).

The eland population in Addo averaged around 100 individuals and these were encountered by lions as frequently as expected based on their total relative abundance (Fig. 1). Like buffalo, the eland population was made up of one large herd of up to 80 individuals plus ∼ five small bachelor herds and, if we consider the population as six herds, then we see lions encountered eland far more than expected based on their herd abundance (Jacobs' index  = 0.83±0.06). Lions hunt eland far more than expected based on their encounter rate, but only kill them in proportion to the number of hunting attempts (Fig. 1). There were not enough hunts of eland during 96 hour follows to calculate hunting success, however lions non-preferentially killed eland in Addo, as they do elsewhere (Fig. 1).

The 280 hartebeest in Addo were encountered by lions far more frequently than expected based on their relative abundance (Fig. 1). Lions hunted hartebeest far less than expected based on this high encounter rate and killed them as frequently as expected based on the number of hunts (Fig. 1). Lions had the lowest hunting success of hartebeest amongst the most frequently hunted species, although this improved in the second year of the study (Fig. 2). Hartebeest were non-preferentially preyed upon in Addo, as they do elsewhere (Fig. 1).

The ostrich population of Addo varied between 180 and 260 over the study period. These individuals were encountered as frequently as expected, however they were hunted far less frequently than expected based on the number of encounters, even though the number of kills were far more frequent than expected based on the hunting effort (Fig. 1). There were not enough observed hunts to determine hunting success rate for ostrich, however they were non-preferentially hunted in Addo, whereas they were significantly avoided throughout the rest of the lion's range (Fig. 1).

Addo's elephant population exceeded 300 individuals made up of eight herds and dozens of male groups [8], [9]. Lions encountered elephants far less frequently than expected based on their abundance and never hunted or killed them on 96 hour follows (Fig. 1). They were observed being hunted at other times however. This led to complete avoidance of elephants by Addo's lions and highly significant avoidance of elephants throughout their range (Fig. 1).

Lions encounter preferred prey more frequently than expected (Table 1). Conversely, they hunt avoided prey less frequently than expected (Table 1).

**Table 1:** Summary of the response of lions to each facet of the predatory behavioural sequence for preferred, non-preferred and avoided prey in Addo Elephant National Park.

Sequence of behaviour	Preferred prey	Non-preferred prey	Avoided prey
Abundance to encounters	+	∼	∼
Encounters to hunts	∼	∼	−
Hunts to kills	∼	∼	∼

Preferred prey are buffalo and zebra; non-preferred prey are those species killed in accordance with their abundance (kudu, warthog, eland and hartebeest); and avoided prey are ostrich and elephant. Symbols refer to behaviours occurring more frequently than expected (+), mixed response amongst the group or as expected (∼), and less frequently than expected (−).

## Discussion

Both buffalo and zebra were preferentially preyed upon in Addo. This preferential predation arose through a high encounter rate (zebra and buffalo herds) and a high hunting rate (buffalo). Hence, preferential predation appears to be initiated early in the predatory behavioural sequence in a stalking predator like lions, as it is in coursing African wild dogs [7]. The open vegetation of the areas inhabited by zebra in the park probably mean that they detect lions before hunts were initiated, which led to a lower than expected hunting rate of zebras.

Hunting success is not highest for the preferred prey species (Fig. 2) suggesting it is actions earlier in the predatory sequence that drive preferential predation through either foraging in areas likely to yield encounters with preferred prey species or hunting those prey more frequently when they are encountered. Once a lion (and probably most predators) initiates a hunt on an animal, evolutionary adaptations probably determine the outcome.

Our results agree with data on African wild dogs, which showed that their prey preferences were the result of a series of preferential decisions throughout the predatory behavioural sequence [7], [10]. African wild dogs have far greater opportunity to actively select prey as they are coursing hunters and their hunts can last several minutes over several kilometres [6], [7], [11], [12], [13], [14], [15]. Conversely, lions are stalking, ambush predators [2], [16], [17] and consequently are expected to have far less opportunities to actively decide what to hunt. Our results show that by increasing encounter rates (via deciding to forage in habitats rich in preferred prey) and, probably, deciding to hunt preferred prey more frequently than non-preferred or avoided prey, lions are actively and cognitively deciding on what to hunt.

Lions had a higher than expected encounter rate with warthog and the highest hunting success rate for this species (Fig. 1 & Fig. 2). Yet warthog in Addo and elsewhere are non-preferentially preyed upon probably because lions hunt them less frequently than expected based on their encounter rate. The high encounter rate is probably due to similar habitat use that may be an artifact of lions targeting other species while hunting in that habitat, but is also confounded by the strict diurnality of warthogs [18], which means lions encounter them when they are less likely to be hunting [2]. The high hunting success is probably due to opportunistic predation when lions hunt warthog when they are easy to kill.

The lions' hunting success rate improved from the first year to the second year of the study for all species except buffalo (Fig. 2). The prey species in Addo had not experienced a large predator for over 100 years [19] and would have been highly predator naïve. It could be expected that this would have resulted in a higher hunting success rate of the lions in the first year of the study than the second. Elsewhere, prey species have learnt appropriate vigilance within months of lion reintroduction [20] and within a generation of wolf *Canis lupus* reintroduction [21]. Conversely, the lions came from South Africa's Kalahari Gemsbok National Park as sub-adults [22] and so would have been inexperienced at hunting novel prey such as kudu, warthog and buffalo. This may have reduced their hunting success rate initially and experience may have led to the higher hunting success rate in the second year. The fact that hunting success improved in the second year after the reintroduction suggests that prey species are faster at adapting to novel predators than lions are to novel prey. This contrasts with bank voles *Clethrionomys glareolus* where anti-predator behaviour appears innate [23], however supports the findings on larger mammals that rapidly reinstated appropriate anti-predator behaviour following wolf *Canis lupus* [21] and lion reintroduction [20].

It could be argued that small sample size may limit the broader conclusions of this study, however these results came from observations of six unrelated lions living in two to five groups reintroduced to a novel environment with novel prey, so their actions are likely to reflect characteristics of the species as a whole. Furthermore, it is highly unusual to be able to monitor individual lions at such a high level of detail and few other studies have been able to achieve this [17]. Finally, the fact that the prey preferences of Addo's six lions corresponds to the prey preferences of lions determined from 22,684 kill records from throughout the lion's distribution supports the validity of our conclusions [1].

There are other factors that may influence the decision making process in stalking predators. Hunger level may influence the decision whether to attempt a hunt, however there was no relation between hunger and hunting effort in Addo's lions, although this may have been due to methodological problems [24]. Group size and distance traveled (energetic costs) may also affect the decision to hunt [25]. Conversely, Addo's lions were reintroduced and were naïve predators of a novel prey community and this may have affected their predator behaviour. This seems unlikely given the prey were similarly naïve to large predators [26] and the hunting success rate of Addo's lions was similar to that of lions elsewhere [2].

The lions reintroduced to Addo's dense thicket environment came from the open, arid Kalahari ecosystem after an absence of over 100 years. Both predators and prey rapidly altered their behaviour to contend with these new challenges and there is little evidence to date that the reintroduction programme has caused a predator-prey imbalance.

## Materials and Methods

We studied lion predatory behaviour in the 134 km^2^ Main Camp section of South Africa's Addo Elephant National Park (Addo) where six radio collared lions (four male, two female) were reintroduced by the South African National Parks (SAN Parks Project Approval No. 2004-03-01GKER) in 2003 after an absence of over 100 years [22]. These lions hunted alone, in pairs, and in groups of three, four and five as the social organization of Addo's lion population evolved from one group of five, to two paired male coalitions and solitary females. Thus, while this study reports on the behaviour of six lions, these lions were faced with a variety of hunting circumstances in a completely novel environment with a novel suite of prey species that, we believe, reflects the decisions faced by lions throughout their distribution. Indeed, few other studies have been able to study six unrelated lions in as much depth as has been possible for Addo's lions [24].

Addo is situated in the Eastern Cape Province (33°30′S, 25°45′E) and is approximately 70 km north of Port Elizabeth. The original vegetation of Addo was dominated by spekboom *Portulacaria afra* as part of the densely vegetated Thicket Biome [27]. This vegetation has been transformed in sections of Addo to open grasslands and thicket clumps following agricultural land uses prior to incorporation into the rapidly expanding park.

Prey species abundances at Addo have been published previously [28]. Buffalo and zebra are the only significantly preferred prey species of lions in Addo, however the zebra population was less than 40 for the duration of the study meaning that rarity in the environment might protect them from targeted preferential predation because it was a sub-optimal strategy [1], [28]. Warthog *Phacochoerus africanus* and kudu *Tragelaphus strepsiceros* are also present and are generally killed more frequently than expected in the thicket biome [28], however eland *Tragelaphus oryx* and hartebeest *Alcephalus busephalus* are killed in accordance with their availability [1]. Conversely, ostrich *Struthio camelus* and elephant *Loxodonta africana* are universally avoided by lions [1], despite some prides hunting them frequently [29], and in Addo these species would be expected to be avoided by lions.

Data on lion hunting behaviour was collected during 16 continuous follows of focal animals that occurred twice per season between summer (December) 2003 and spring (November) 2005. These continuous follows involved following the focal animal continuously for periods of up to 96 hours [30], [31], [32], although we modified this by leaving the focal lion for periods of less than two hours during the hottest times of the day. Two follows were cut short by six hours as a kill was made within 24 hours of the intended completion time. Follows were conducted from a 4WD vehicle. A night vision scope was initially used however this was replaced by spotlights after the first season because this resource was redirected elsewhere within SAN Parks. When prey were encountered, spotlights were extinguished and the outcome of hunts was determined by sound or moon/starlight.

We recorded an encounter with prey if the prey individual or group was detectable (visually or audibly) to us and if there were no obvious impediments to detection by the focal lion. We counted the number of encounters with groups rather than the number of individuals in each group encountered because we were unable to accurately count group sizes in the dense, thicket vegetation. We defined hunts as characteristic behaviours that involved focused attention on a potential prey item, stalking toward the prey individual, and ultimately charging [2]. This complete behavioural repertoire was not necessary for us to define a hunt, as opportunistic hunts sometimes lacked stalks, such as when lions lay in thick vegetation or by waterholes, or even the initial focus on the potential prey item when the lions awoke, detected prey and then charged [2], [33], [34], [35]. Where a hunt ended in dense vegetation and the lions remained inactive for more than two hours, we walked in to the carcass to confirm the identity of prey.

Annual estimates of the prey community were made by standardized aerial censuses using helicopters and several observers. These data have been published elsewhere [28]. Aerial counts are biased toward larger prey, however this bias against smaller items is generally alleviated in preference assessments by the use of relative measures of abundance. The adult lion population remained at six throughout the study. Spotted hyaenas were also present, however these did not appear to affect lion predation rates as they ingested more meat than any other lion population recorded, except those in Etosha National Park, Namibia [24], and even single lioness activity patterns appeared unaffected by hyaena presence [36]. The solitary leopard that was reintroduced in 2004 [37] is also unlikely to have affected lion predator behaviour.

We used Jacobs' selectivity index [38] to measure the preference for certain species during each phase of the predatory behavioural repertoire. Although there are numerous selectivity indices [39], Jacobs' index is one of the better ones as it minimizes several of the problems that afflict many other indices, such as non-linearity, bias to rare food items, increasing confidence intervals with increasing heterogeneity, being unbound or undefined, and lacking symmetry [40], [41], [42]. The formula for Jacobs' index is:
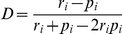
where *p_i_* is the relative proportion of the previous predatory behavioural phase (abundance, encounter rate, hunt) and *r_i_* is the relative proportion of the latter predatory phase (encounter rate, hunt, kill). We analysed buffalo and eland abundance based both on individual counts and our estimate of number of mixed sex and bachelor herds. This was not conducted for other species, as buffaloes and eland exist in large herds of up to 100 individuals and so the discrepancy between the number of individuals and herds is greatest for them. We considered Jacobs' index values >0.2 as implying preference and <−0.2 as implying avoidance, with values between these two figures indicating a behaviour is performed as frequently as expected.
